# In Situ Raman Spectroscopy as a Tool for Structural Insight into Cation Non-Ionomeric Polymer Interactions during Ion Transport

**DOI:** 10.3390/polym10040416

**Published:** 2018-04-09

**Authors:** Krzysztof Artur Bogdanowicz, Domenico Pirone, Judit Prats-Reig, Veronica Ambrogi, José Antonio Reina, Marta Giamberini

**Affiliations:** 1 Departament d’Enginyeria Química, Universitat Rovira i Virgili, Av. Països Catalans 26, Campus Sescelades, 43007 Tarragona, Spain; mimmopirone@gmail.com (D.P.); jupratsreig@gmail.com (J.P.-R.); 2 Military Institute of Engineer Technology, 136 Obornicka Street, 50-961 Wroclaw, Poland; 3 Dipartimento di Ingegneria dei Materiali e della Produzione, Università di Napoli ‘Federico II’, Piazzale Tecchio 80, 80125 Napoli, Italy; veronica.ambrogi@unina.it; 4 Departament de Química Analítica i Química Orgànica, Universitat Rovira i Virgili, Carrer Marcel·lí Domingo s/n, Campus Sescelades, 43007 Tarragona, Spain; joseantonio.reina@urv.cat

**Keywords:** in situ ion transport, Raman spectroscopy, cation permselectivity

## Abstract

Low-modified liquid-crystalline polyether (CP36), as a model compound, was synthesised with the purpose of preparing a membrane with columnar ionic channels. A free-standing cation permselective biomimetic membrane was successfully prepared and found to have channels made of polymeric columns homeotropically oriented, which was confirmed in X-ray diffraction (XRD) analysis. A first insight into a real-time interaction between two selected cations: H^+^ and Na^+^, and polyether during transport through the polymeric membrane was demonstrated using joined chronoamperometry and Raman spectroscopy techniques. Raman studies unveiled the possibility for smaller protons to bypass the usual ionic pathway via polyetheric chain and use outer part of ionic channel for conduction thanks to ester bonds.

## 1. Introduction

Progress in modern civilisation has been marked by ever-increasing energy and power requirements, and clean water consumption. Since the industrial revolution, the majority of needs are provided mainly by non-renewable resources. This has resulted in severe air and water pollution and contributes significantly to global warming and the decline of potable water assets. Due to this tendency, alternative technologies for energy and water production have received a great deal of attention in recent years [1,2,3]. Therefore, environmentally-friendly electrodialysis, artificial photosynthesis, and polymer electrolyte membrane fuel cells seem appealing as an alternative to conventional technologies. Moreover, they are silent and non-polluting, and their emissions are ultra-low [4,5,6,7].

The key elements in such systems are ion exchange membranes (IEMs) which play a vital role as a cation or anion conductor, an electrical insulator between anode and cathode, and a separator between the fuel (methanol) and oxidant (oxygen or air). IEMs can be made either from pure polymer membranes or from composite membranes, where other materials are embedded in a polymer matrix [8,9,10,11,12].

In the literature, are some examples of non-ionomeric materials able to conduct ions, mainly ceramic oxides, like CeO_2_ doped with other metals for example: Sr, Fe, or Gd [13]. Other ceramic proton-conducting materials are anhydrous ruthenium oxide doped with Ti_4_O_7_ nanoparticles, and sulphonic acid functionalised silica that can be used as a substitute for typical polymeric IEMs in a polymer electrolyte fuel cell (PEFC) [14]. Alternatively, a strategy including a metal oxygen framework (MOF)—polymer composite membranes was reported, where enhancement of proton conductivity, supported by tertiary amine as carriers, was observed at low-humidity [15].

Liquid crystals (LCs) as ion transporting materials became an emerging and promising field thanks to their versatility to adapt numerous diversity of assemblies and self-assembled arrays [16,17,18,19]. Those systems are able to mimic biological structures, forming nanochannels that convey ions. The versatility of LCs can be programmed and changed: for example, their orientation can be induced by shearing or combination of thermal treatment with ultra-violet (UV)-light exposure [20,21].

One of the important factors that need to be considered is the interaction between active material in the membrane and ions passing through, exerting an influence on the transport performance. Most of the studies were performed on materials which were composed of ions and used electrochemical impedance spectroscopy (ESI) for ion transport measurements and Raman spectroscopy to record the behaviour of ions in the matrix, or cross-over effect of water and fuels [22,23,24,25].

However, in the case of non-ionomeric membrane, the key issue is to register cation-polymer interactions forcing mainly cations to the flow across the membrane without the influence of water and anions.

Non-ionomeric membranes based on liquid-crystalline dendrimers grafted to polyether and polyamines have proven their ability to transport cations across the membrane. The ionic conductivity occurs due to the polymer specific columnar assembly gained through a self-organization process from an isotropic melt into liquid-crystalline columnar mesophases [26,27,28,29].

In this case, the mesogenic dendritic groups are responsible for self-organization via an exo-recognition process and form outer layers of the column, whereas the polymer backbone forms the inner part, responsible for the ion transport [17,30].

It was suggested that cation transport in liquid-crystalline dendrimer-grafted polyamines could occur thanks to coordination and solvation-disolvation along the polymer main chain; however no direct proves were reported [31].

In this work, Raman spectroscopy was applied in order to give a first insight into the permeaselective cation transport of an oriented biomimetic membrane. Based on our previous experience, as a model system we chose a poly(epichlorohydrin-*co*-ethylene oxide) modified in 36% with a tapered mesogenic group, which exhibited the desired properties: liquid crystalline behaviour, the ability to self-assemble into a columnar structure, formation of a free-standing membrane with homeotropically oriented channels, and cation permeaselectivity. Those characteristics make this polymer a suitable candidate as a model compound for the observation of polymer response to electrically induced ion transport. To the best of our knowledge, we used chronoamperometry to induce ion flow in a home-made Raman cell for the first time, to have a closer insight on cation-non-ionomeric polymer interactions during ion transport. In the experiment, two selected cations, H^+^ and Na^+^, were used as transported cations through a polymeric membrane.

## 2. Materials and Methods

Materials: Inorganic and organic compounds were provided by Sigma-Aldrich (Darmstad, Germany) and Fisher Scientific (Loughboroug, UK), and used as received. For all experiments that required water or aqueous solutions, Milli-Q water was used. Poly(epichlorohydrin-*co*-ethylene oxide) (P(ECH-*co*-EO), M_w_ = 5.01 × 10^5^ determined by gel permeation chromatography) was used as received.

**Figure 1:**
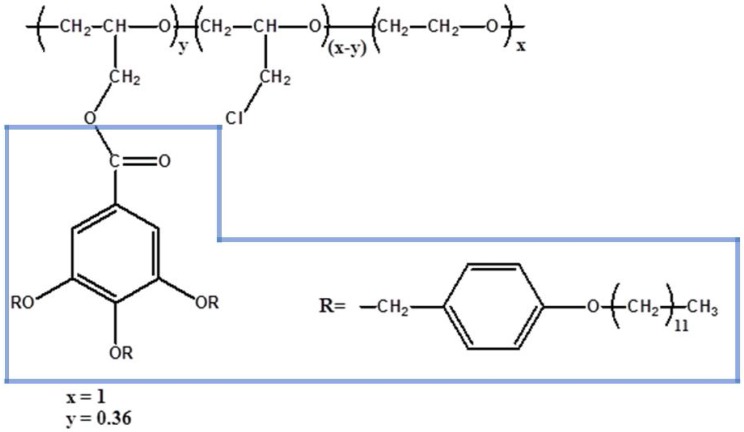
Chemical structure of CP36. In blue TAP group is marked.

Synthesis of polymer: Modified poly(epichlorohydrin-*co*-ethylene oxide) (CP36) was synthesised according to [28]. The polymer structure is reported in Figure 1. The Detailed CP36 polymer description is present in Appendix A: Raman spectroscopy bands assignment, ^1^H and ^13^C NMR data.

Membrane preparation: 30% (*w*/*w*) polymer solution was prepared in tetrahydrofuran (THF). Once it was completely homogeneous, it was spread over a fluorinated ethylene propylene (FEP) sheet support and then the membrane was obtained by immersion precipitation in a water coagulation bath. The membrane was kept for approximately 15 min in the water bath to assure the exchange of the solvent (THF) and the nonsolvent and the final demixing process, which takes place when the composition of the polymer solution becomes metastable. Once obtained, the polymeric membrane was dried at room temperature overnight.

Thermal treatment (baking process): Proper membrane orientation was obtained by means of a baking process, as described in our previous work [28]. In this case, the optimum baking process consisted of heating the membrane to 80 °C at 10 °C/min, keeping it at this temperature for 10 min and finally cooling to room temperature at −0.1 °C/min.

Contact Angle (CA): The static contact angles with water on a membrane surface were measured with a Dataphysics (Filderstadt, Germany) OCA 15EC contact angle instrument equipped with a motorised pipette and deionised water as the probe liquid. The contact angle was measured immediately after placing the water drop (3 μL) on the membrane surface. The measurements were repeated using different areas of the film: for each test reported, at least three drops of water were used.

X-ray Diffraction (XRD): For low 2Θ range, XRD measurements were performed with a Bruker-AXS (Billerica, Massachusetts, USA) D8-Discover diffractometer equipped with parallel incident beam (Gӧbel mirror), vertical Θ-Θ goniometer, an XYZ motorised stage, and with a General Area Detector Diffraction System (GADDS). Samples were placed directly on to a low background Si(510) sample holder for reflection analysis. An X-ray collimator system close-to-the-sample allows to analyse areas of 500 mm. The X-ray diffractometer was operated at 40 kV and 40 mA to generate Cu_Kα_ radiation. The GADDS detector was a HI-STAR (multiwire proportional counter of 30 × 30 cm with a 1024 × 1024 pixel) placed at 30 cm from the sample. The X-ray beam hit the sample at 0.5° of incidence. The collected frame (two-dimensional (2D) XRD pattern) covers a 2q range from 0.9 up to 9.2°. The diffracted X-ray beam travels through a He beam path (Small Angle X-ray Scattering attachment) to reduce the air scattering at low angles. The direct X-ray beam is stopped by a beam stop placed directly on the detector face. The exposition time was of 300 s per frame. The resulting frames were both gamma integrated to obtain a 2Θ diffractogram and 2Θ integrated to obtain an azimuthal intensity plot. In the case of membranes, XRD experiments were performed on both sides and no differences could be detected.

Differential Scanning Calorimetry (DSC): Thermal transitions were detected with a Mettler-Toledo (Greifensee, Switzerland) Differential Scanning Calorimeter mod. 822 in dynamic mode at a heating or a cooling rate of 10 °C/min in cycle: First heating from −100 °C to 140 °C, followed by cooling to −100 °C and second heating to 300 °C. Nitrogen was used as the purge gas. The calorimeter was calibrated with an indium standard (heat flow calibration) and an indium-lead-zinc standard (temperature calibration).

Scanning Electron Microscopy (SEM): SEM analysis was performed using a FEI Microscopy (Eindhoven, The Netherlands) FEI Quanta 200 FEG in high vacuum mode, using a secondary electron detector and an accelerating voltage ranging between 15 and 20 kV. The samples were fractured by impact after cooling them with liquid nitrogen. In the resulting specimens, a brittle fracture was obtained. Before the analysis, samples were coated with a gold-palladium layer (about 15 nm thick) by means of a Quorum Technologies (Lewes, UK) Emitech K575X sputter coater.

Polarised Optical Microscopy (POM): The clearing temperatures were roughly estimated by POM; textures of the samples were observed with an Carl Zeiss Jena GmbH (Jena, Germany) Axiolab Zeiss optical microscope equipped with a Linkam Scientific Instruments LTD (Tadwort, UK) LinKam TP92 hot stage.

Solution Uptake Test: Weighted membranes were immersed in deionised water at room temperature for 15 to 120 min (measured every 15 min) and finally for 24 h to ensure the membranes were saturated. Detailed description was given in our previous work [32].

**Figure 2:**
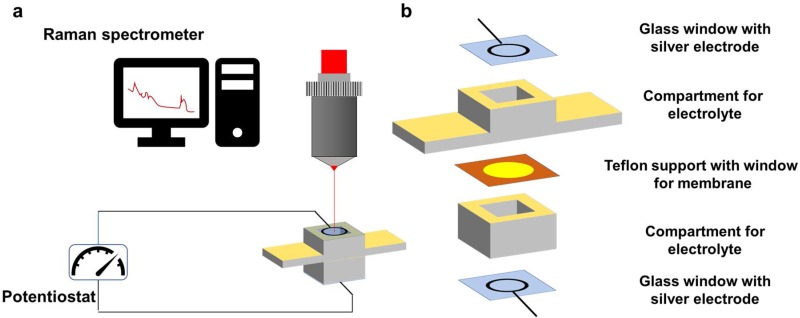
Setup for ion transport experiments (**a**) and the construction of Raman cell (**b**) detailed Raman cell assembly.

Raman Spectroscopy: Raman studies were carried out using a Renishaw (Gloucestershire, UK) Confocal Microscope (Leica DM 2500) with FT-IR (IluminatIR II) and Smith Streamline Raman Imaging module equipped with a 785-nm diode laser (300 mW) was used to acquire the Raman spectra at laser power of 10%. The spectra were recorded in a range from 300 cm^−1^ to 3200 cm^−1^ or 3700 cm^−1^ before the constant potential application, during its application and once the potential was stopped. Time of bleaching before the first spectra accumulation was 360 s. The laser beam was focused at 10 nm below the membrane surface using a confocal microscope to identify the membrane surface, and then automatic micromotors to move the focal point below the surface. The minimum theoretical beam spot (analysed area) due to the objective used, was 1.915 µm^2^. A small scale chronoamperometric cell was designed in order to permit registration of the spectra at the same time that electrolysis was performed. The cell consisted of two compartments with one electrode each, divided by tested membrane (Figure 2). The electrode was a coiled silver wire fixed to the glass window with sufficient space between loops that allowed the beam to be focused onto the membrane surface without any interferences.

Linear Sweep Voltammetry (LSV): Linear sweep voltammetry was performed using Metrohm (Herisau, Switzerland) Autolab PGstat 204 in potentiostatic mode with current ranging (automatic) from 100 mA to 100 μA, a potential range from 0 V to 5 V, step 0.01 V and a scan rate 0.01 V s^−1^. The experimental setup and experimental procedure were described in our previous work [31].

Chronoamperometry: Chronoamperometry was performed using Autolab PGstat 204 with set constant potential of 2, 3 or 4 V. Current response was registered every 1 s during all experiments while Raman spectra was recorded.

## 3. Results and Discussion

**Figure 3:**
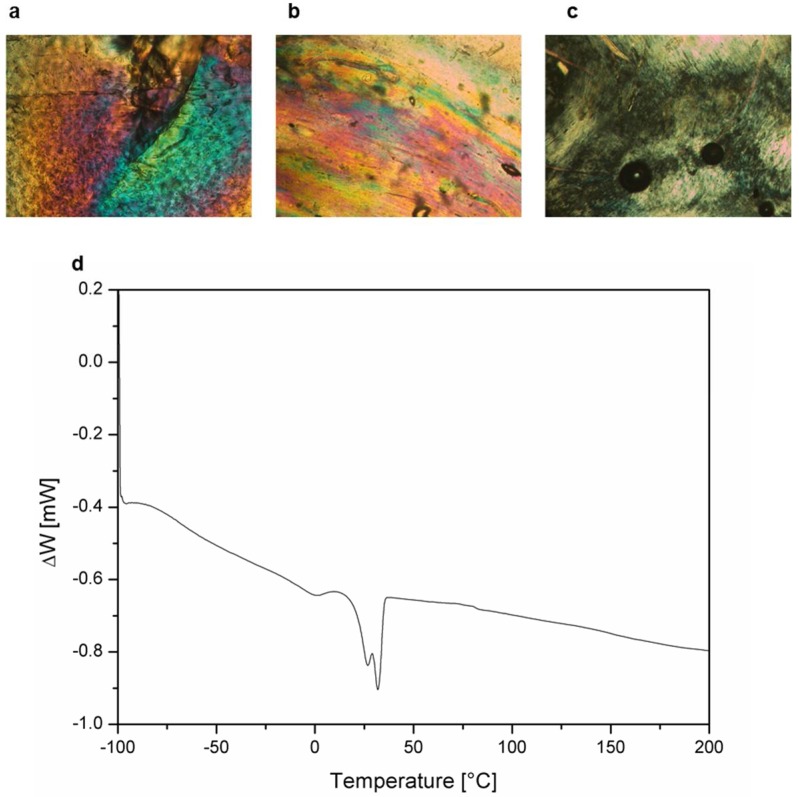
Polarised optical microscopy (POM) images of CP 36 (**a**) at 25 °C, (**b**) at 35 °C, (**c**) at 59 °C and (**d**) Differential scanning calorimetry (DSC) curve for the second heating scan.

The objective of this study was the observation of in situ ion transport in a liquid-crystalline biomimetic membrane using coupled techniques: Raman spectroscopy and chronoamperometry. In this way, we got a first inside observation into the mechanisms of cation transport inside this kind of membrane. To the best of our knowledge, this is the first example of a combined Raman-amperometric experiment, which, in addition, we performed on a novel class of cation transport material. As we have quite recently reported, polyethers and polyamines which bear a side tapered group, self-assemble into a columnar structure and exhibit promising small cation transport without the need for water: this evidence opens new possibilities for application and could overcome the drawbacks of currently used membranes. On the other hand, these findings arouse questions about the mechanism of transport across these novel materials: according to our hypothesis, transport is expected to occur thanks to the interaction of the cations with basic oxygen or nitrogen atoms. Based on our previous work, as a sample for this preliminary study we selected one of the previously reported biomimetic systems, namely modified P(ECH-*co*-EO) with wedge-shaped liquid-crystalline molecule (TAP): as a matter of fact, Bhosale et al. reported quite high degrees of modification, in a range of 57–69% (CP57-69) [33]. These materials could be also easily oriented, formed self-sustaining, mechanically stable membranes and showed good conductivity, comparable to Nafion 117^®^ membranes [28]. For our experiments, we were interested in having higher backbone mobility at room temperature, since this is an important factor which can improve cation transport by lowering internal resistance; on the other hand, our model system needed to keep the columnar ordering of the LC phase, which was due to the presence of bulky TAP group, which was essential for the formation of ion-conducting paths. These characteristics could be successfully combined by lowering the modification degree of P(ECH-*co*-EO) to 36% (CP36): in this way, the clearing temperature of the LC phase became as low as 32 °C (Figure 3). In order to confirm the expected properties and qualities of polymeric membranes, the usual characterization techniques were employed. The microscopic observations under crossed-polarised light (Figure 3a–c) and DSC (Figure 3d) confirmed liquid-crystalline nature of the polymer at 25 °C, as desired, and clearing at 32 °C.

**Figure 4:**
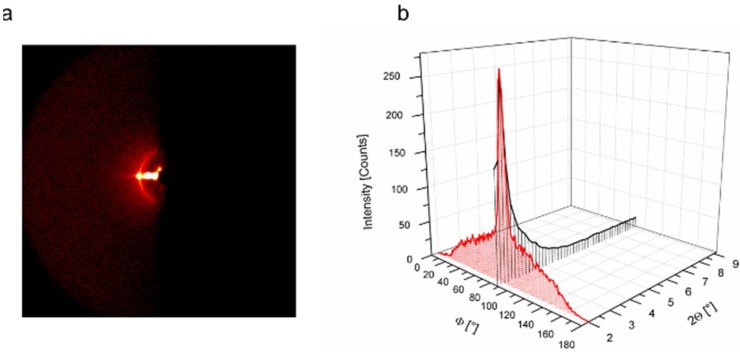
X-ray diffraction (XRD) results for the oriented CP36-based membrane: (**a**) Debye ring, (**b**) 2-theta and azimuthal scale.

Next, CP 36-based membranes were prepared by casting a polymeric solution on a fluorinated ethylene propylene surface (FEP), followed by a thermal treatment. Free-standing polymeric thin films obtained in this way, possessed both a columnar phase and homeotropical-like orientation of the polymeric columns, as desired. The XRD patterns of CP36 (Figure 4) showed just one signal at 2Θ = 2.1°, which indicates columnar formation with a diameter around 4 nm, akin to previously reported CPs with higher modification degrees [28]. Furthermore, the signal in the azimuthal scan at 2Θ = 2.1°, exhibited a peak at 91° with FWHM (Full Width at Half Maximum) equal to 7°, which evidences columnar orientation almost perfectly perpendicular to the surface.

Previously reported CP-based membranes showed hydrophobic surfaces and negligible water uptake; to test whether the low modification degree of CP36 has an impact on membranes’ hydrophobicity, a CA study and a water uptake test were performed; results confirmed the hydrophobic nature of the oriented membranes, giving an average angle of 110°. The water uptake test revealed low water absorption over time, reaching a maximum value of 4 ± 1% after 24 h (Figure S1). This value is in the range observed for other polyethers and polyamines containing TAP as a mesogenic unit [28,29,31].

**Figure 5:**
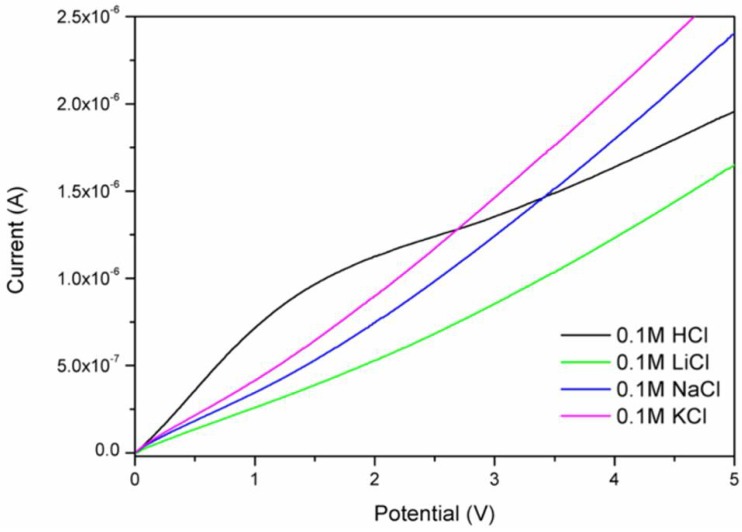
Linear sweep voltammetry (LSV) curves for different 0.1 M electrolyte solutions.

In order to establish the value of applied potential necessary for cation transport and to confirm cation permeaselectivity, linear sweep voltammetry (LSV) measurements were performed. The experiments involved measurements in 0.1 M solutions of electrolyte: hydrochloric acid, lithium chloride, sodium chloride, and potassium chloride. For all cations, the obtained current versus voltage data did not show a linear trend; the curves revealed evidence of a typical three-region division into ohmic, limiting current, and overlimiting regions, as expected. The first inflection, which indicates the beginning of the limiting current region, was found to be approximately at 1.5 V for protons (see Figure 5), which also possessed the highest observed value; hence, 2 V was selected as the starting potential for further experiments to prevent accumulation of cations at the membrane surface [34]. The presence of well-pronounced limiting current sections indicated cation permeaselective transport in the CP36-based membrane. This means that charge transfer in the membrane is mainly influenced by the transported cations [31]. Further, a deeper analysis of LSV resulted in the establishment of preferential cation transport arranged in order of decreasing conductance: H^+^>>K^+^/Na^+^>Li^+^. In this order, potassium and sodium cations did not show a clear preference between them, and occasionally their places were often swapped. This fact can be easily explained due to the ionic nature of both Na^+^ and K^+^ compounds, in contrast with the partial covalent character of Li^+^ compounds [35].

**Figure 6:**
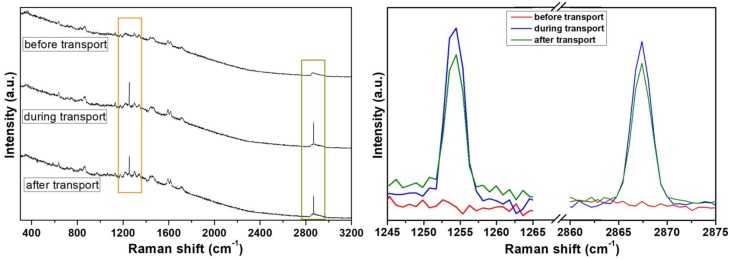
Raman spectra of CP36 in 0.1 M HCl before, during and after transport experiment: full range (**left site**) and onset of changed region (**right site**).

**Figure 7:**
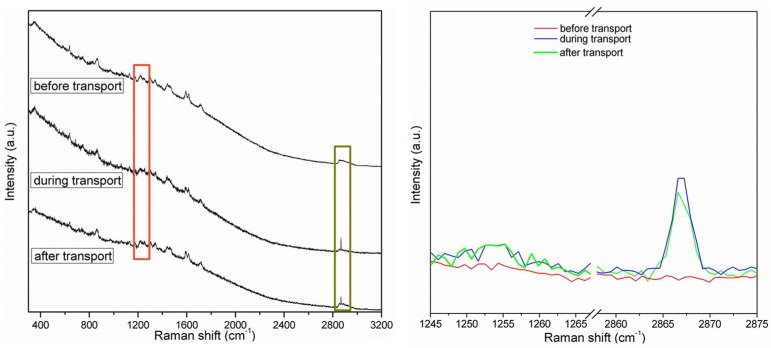
Raman spectra of CP36 in 0.1 M NaCl before, during, and after transport experiment: full range (**left site**) and onset of changed region (**right site**).

**Figure 8:**
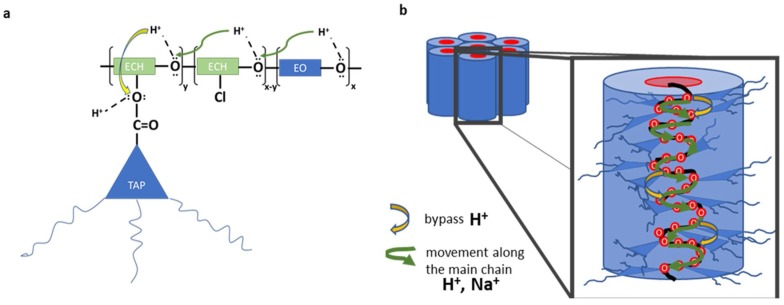
Schematic representation of cation movement inside the ionic channel: (**a**) hopping proton along polymeric chain (ECH—epichlorohydrin and EO—ethylene oxide) and (**b**) ion movement through single column.

For in situ ion-transport studies, a special Raman cell was designed and constructed: it consisted of two compartments with a glass window with silver electrodes on both sides and separated by the polymeric membrane (Figure 2). Raman spectra were recorded deploying 0.1 M solutions of hydrochloric acid or sodium chloride before-, during-, and after constant potential had been applied, to register the structural response of the CP36 polymer to cation transport. Detection of steady current flow of an order of 10^−7^ A during spectra accumulation, assured ion transport in the experimental conditions. Significant changes in the spectra were observed regarding the intensity of bands at 2870 cm^−1^ and at 1250 cm^−1^ for proton, while applying potential through the cell (Figure 6). The band at 2870 cm^−1^ is characteristic of C–H stretching vibration of methylene group of ether unit from the polymer main chain, [36] whereas the band at 1250 cm^−1^ corresponded to the stretching vibration of the (O=C)–**O**–C ester lateral group [37]. On the other hand, in the case of the sodium cation, only the band at 2870 cm^−1^ showed increased intensity (Figure 7). This suggests, in the case of a smaller cation such as H^+^, the proximity of an additional coordinating site in the lateral position of the polymer backbone, being in the same distance as the next etheric oxygen in main chain, is involved in its transport (Figure 8a), while this does not occur in the case of Na^+^.

**Figure 9:**
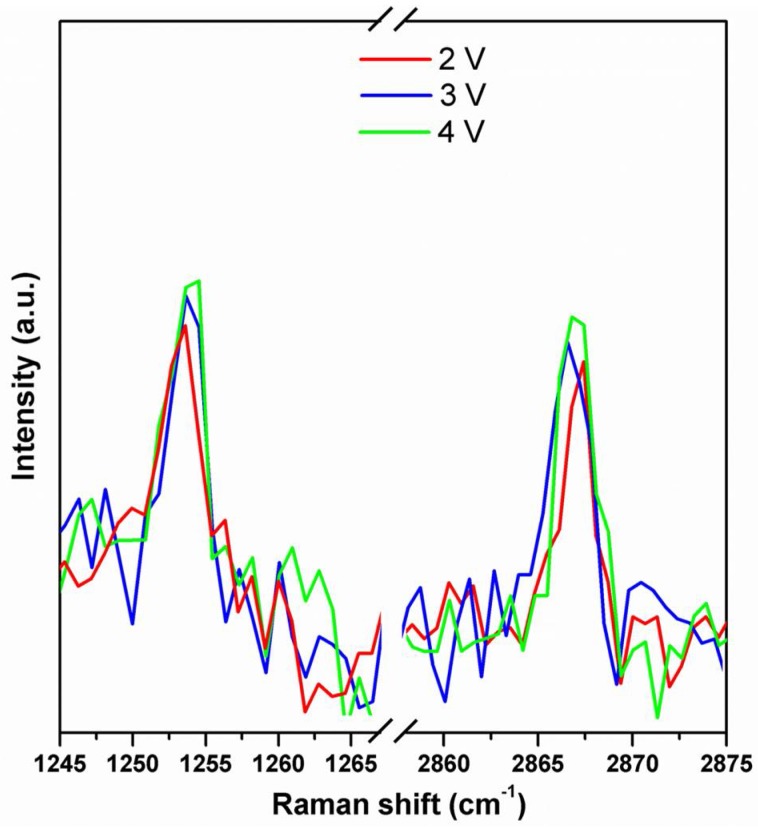
Onset of changed Raman spectra regions of CP36 in 0.1 M HCl during the transport experiment at 2 V, 3 V and 4 V.

In other words, protons can hop not only between the oxygen atoms of main chain, but can also use the additional coordinating position of the ester groups in the side chains and hop to another closest available coordination site, which most likely shortens the passageway through the membrane. As a result of such behaviour, as far as we can tell, the cation could hop from the ester TAP connection to the closest vicinal acceptor location of another TAP group or polyetheric main chain (Figure 8b). Additional experiments for the proton cation with two other values of applied potential, 3 V and 4 V respectively, were performed. In the case of protons, measurements disclosed the expected increase of the current value due to higher voltage, giving values fluctuating around 1.5 × 10^−6^ A and 3.0 × 10^−6^ A for 3 V and 4 V, respectively, whereas for 2 V, the current value was 0.3 × 10^−6^ A. As anticipated, an increase in cation passage provoked an increase in the intensity of two bands at 1250 cm^−1^ and 2870 cm^−1^; however, the scale of increment observed in Raman spectra was not proportional to applied voltage, i.e., roughly 6% and 12% for 3 V and 4 V, respectively (Figure 9). This can be interpreted as an increased proton flux as a consequence of increased external potential, which did not affect proton concentration inside the membrane significantly. In all experiments, the influence of water on cation transport was discarded, since the membrane exhibited low water uptake values of 4 ± 1%; as a matter of fact, the range between 3200 cm^−1^ and 3700 cm^−1^ in Raman spectra, characteristic of OH stretching region of water, showed no significant changes.

**Figure 10:**
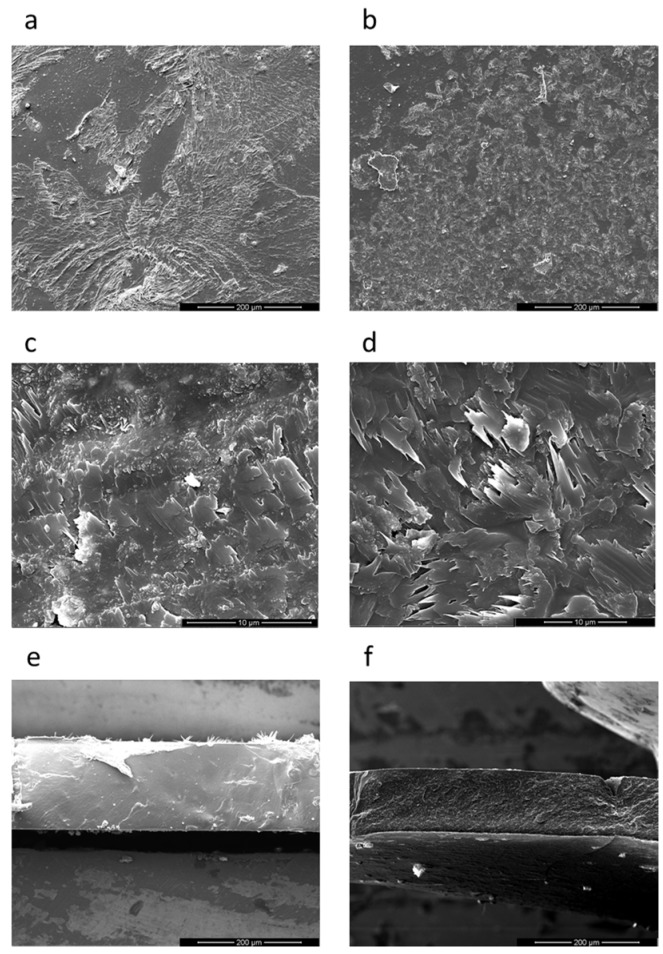
Scanning electron microscopy (SEM) images of the surface (**a**–**d**) and cross section (**e**,**f**) of the membrane: pristine membrane (**left**) and membrane after the chronoamperometry experiment (**right**).

The surface and cross-section of polymeric films before and after chronoamperometry (five cycles of 30 min) observed by SEM (Figure 10) were very similar. This puts into evidence that no mechanical damage was caused by aggressive experimental conditions. Furthermore, in Raman spectra obtained for a membrane after the fifth cycle of chronoamperometric experiments no significant changes in the structure of the polymer were observed, compared to fresh membrane (Figure S2). As well as the current evolution recorded during transport experiments was kept at the level of 10^−7^ A for all cycles (Figure S3).

## 4. Conclusions

In this study, in situ Raman spectroscopy coupled with an amperometric experiment was used for the first time to have a first insight into permeaselective cation transport in an oriented biomimetic membrane. As the study case poly(epichlorohydrine-*co*-ethylene oxide) was modified in 36% of cases, the taper group, exhibiting desired characteristics, and proton and sodium cations, were selected. It was found that the polyetheric backbone is mainly involved in cation conduction, as previously suspected, and this was observed as a change in the intensity of the band at 2870 cm^−1^. Surprisingly, in case of proton transport, an additional coordination site coming from the lateral ester group actively interacts with a proton during its transport, as identified by an intensity change of the band at 1250 cm^−1^. Further, it was confirmed that cation transport in the membrane had not been influenced by the presence of water. Though the role of ester oxygen was confirmed, variations due to different modification degrees cannot be ruled out and this aspect will be deepened in our forthcoming studies. All these findings could justify the use of such liquid-crystalline polymers as a suitable material to prepare ion-conducting membranes for applications, such as electrodialysis or polymer electrolyte fuel cells. Our future studies will be focused on further understanding of the behaviour of these materials, for instance, ion transfer from solution to the hydrophobic surface and its influence on the general membrane performance.

## Supplementary Materials

The following are available online at http://www.mdpi.com/2073-4360/10/4/416/s1, Figure S1: Mass evolution during water uptake test for oriented CP36 membrane at room temperature in water; Figure S2: Raman spectra of fresh membrane (red) and after 5th cycle of chronoamperometry; Figure S3: Current evolution during transport experiments for 1st, 3rd and 5th cycle.

## Appendix A

Copolymer structure was confirmed by ^1^H and ^13^C NMR in solution, and further investigated by Raman spectroscopy:

**^1^H NMR** (400 MHz, TCE-d_2_) δ ppm: 7.4 (8 H, aromatic), 6.9 (6 H, aromatic), 5.00 (6 H, ArO-C**H**_2_-Ar), 4.4 (>CH-C**H**_2_-O-TAP), 3.9 and 3.6 (mainchain and Ar-O-C**H**_2_- aliphatic), 1.8 (6 H, Ar-O-CH_2_-C**H**_2_-CH_2_-), 1.2 (54 H, -C**H**_2_- aliphatic), 0.9 (9 H, C**H**_3_ aliphatic).

**^13^C NMR** (100 MHz, TCE-d_2_) δ ppm: 165.82 (-O-(**C**=O)-Ar), 158.85 (**C**_aromatic_-O-Aliph), 152.48 (**C**_aromatic_-O-CH_2_-Ar, meta), 142.27 (**C**_aromatic_-O-CH_2_-Ar, para), 130.11, 129.22, 128.12, 124.73, 114.33, 113.98, 108.63 (-(O=C)-**C** aromatic), 78.48 (>**C**H-CH_2_-O-TAP), 74.57 (>**C**H-CH_2_-Cl), 70.79, 70.12- 68.90 (-**C**H_2_- mainchain), 67.90 (Ar-O-**C**H_2_- aliphatic), 64.49 (-**C**H_2_-O-TAP), 58.22, 43.83 (-**C**H_2_-Cl), 31.81, 29.57, 25.95, 22.62, 18.40, 14.12.

**Raman** shift cm^−1^: 3075 (ν CH_r_), 3019 (ν CH_r_), 2938 (ν as CH_2_), 2853 (ν_s_ CH_2_), 1716 (ν C=O), 1615 (ν Ph), 1595(ν Ph), 1458 (ν Ph), 1437 (ν Ph), 1383 (δ CH_2_), 1373 (δ CH_2_), 1339 (ω CH_2_), 1301 (ω CH_r_), 1252 (ν (C=O)-O), 1218 (δ CH_r_), 1176 (δ CH_r_), 1128(δ CH/ν COC), 1087 (ν COC), 1064 (δ CH_r_), 1015 (δ CH_r_/ν CO), 974 (γ CH_r_), 929 (γ CH_r_), 891 (γ CH_r_/ν COC), 863 (ρ CH_2_), 826 (γ CH_r_), 745 (γ CH_r_/CH_2_-Cl), 711 (ρ CH_r_/skel. CH_2_), 637 (δ Ph), where: ν– stretching vibrations, δ– in-plane deformation, γ–out-of-plane deformation, ω—wagging, ρ—rocking, Ph—phenyl, CH_r_—CH group in phenyl ring.

Copolymer modification degree was determined by ^13^C NMR at room temperature in deuterated tetrachloroethane (TCE-d_2_), by comparing the areas of the carbon of methanetriyl group (>CH-) of epichlorohydrin unit in the modified monomeric units at 78.5 ppm and the carbon of methanetriyl group (>CH-) in the unmodified monomeric unit at 74.6 ppm.
