# Supplementary material for: In Situ Raman Spectroscopy as a Tool for Structural Insight into Cation Non-Ionomeric Polymer Interactions during Ion Transport
Source: Polymers (Basel). 2018 Apr 9;10(4):416. doi: 10.3390/polym10040416 (PMC6415221; doi:10.3390/polym10040416)
Supplement: Supplementary file 1 [file polymers-10-00416-s001.pdf]

Supplementary Materials

In situ Raman spectroscopy as a tool for structural insight into cation- non-ionomeric polymer interactions during ion transport

Krzysztof A. Bogdanowicz[1,2,*], Domenico Pirone [1,3], Judit Prats-Reig [1], Veronica Ambrogi [3], José A. Reina [4] and Marta Giamberini[1,]**

1 Departament d’Enginyeria Química, Universitat Rovira i Virgili, Av. Països Catalans 26, Campus Sescelades, 43007 Tarragona, Spain; jupratsreig@gmail.com (JP-R)
2 Military Institute of Engineer Technology, 136 Obornicka street, 50-961 Wroclaw, Poland
3 Dipartimento di Ingegneria dei Materiali e della Produzione, Università di Napoli ‘Federico II’, Piazzale Tecchio 80, 80125 Napoli, Italy; mimmopirone@gmail.com (DP), veronica.ambrogi@unina.it (VA)
4 Departament de Química Analítica i Química Orgànica, Universitat Rovira i Virgili, Carrer Marcel·lí Domingo s/n, Campus Sescelades, 43007 Tarragona, Spain; joseantonio.reina@urv.cat
* Correspondence: bogdanowicz@witi.wroc.pl; Tel.: +48-(71)-347-4411
** Correspondence: marta.giamberini@urv.cat; Tel.: +34-977-558-174

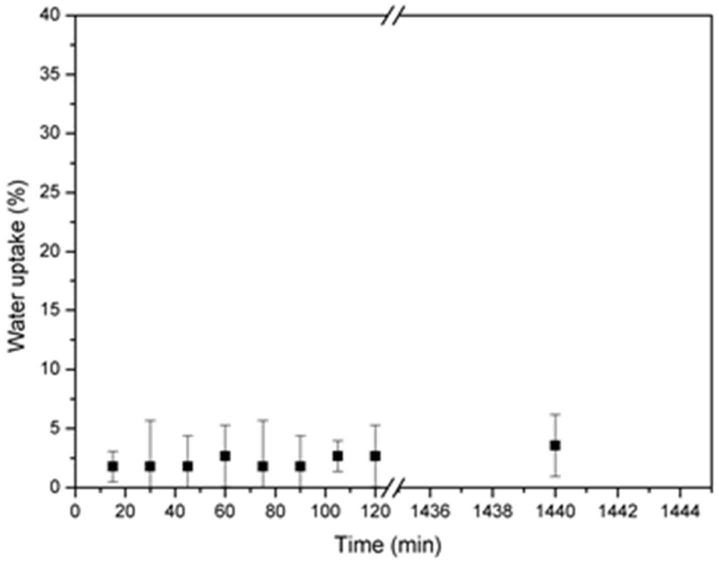

Figure S1. Mass evolution during water uptake test for oriented CP36 membrane at room temperature in water.

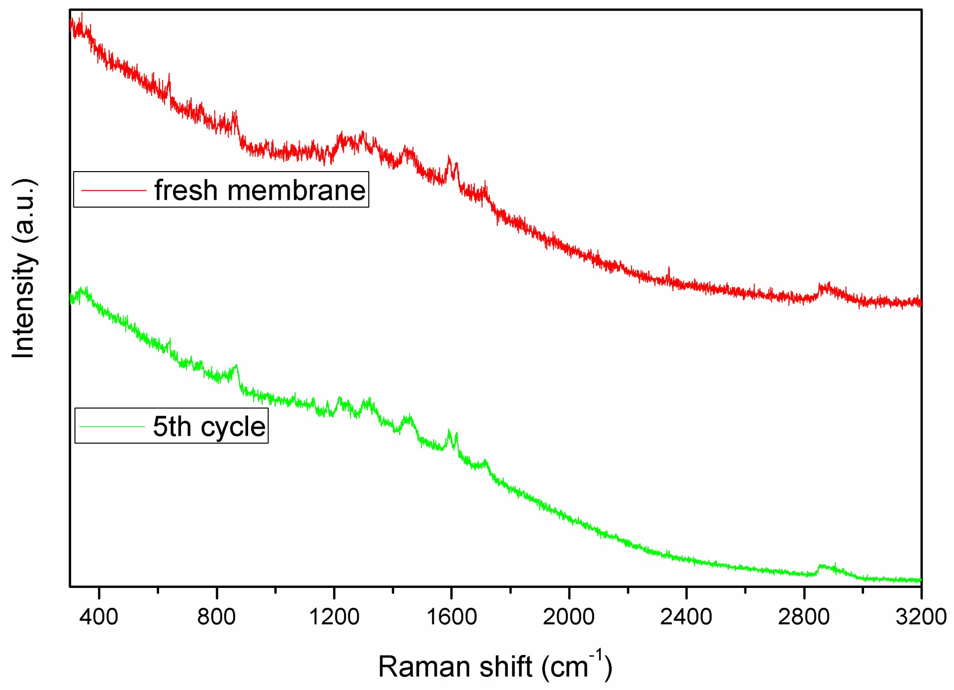

Figure S2. Raman spectra of fresh membrane (red) and after 5th cycle of chronoamperometry (green).

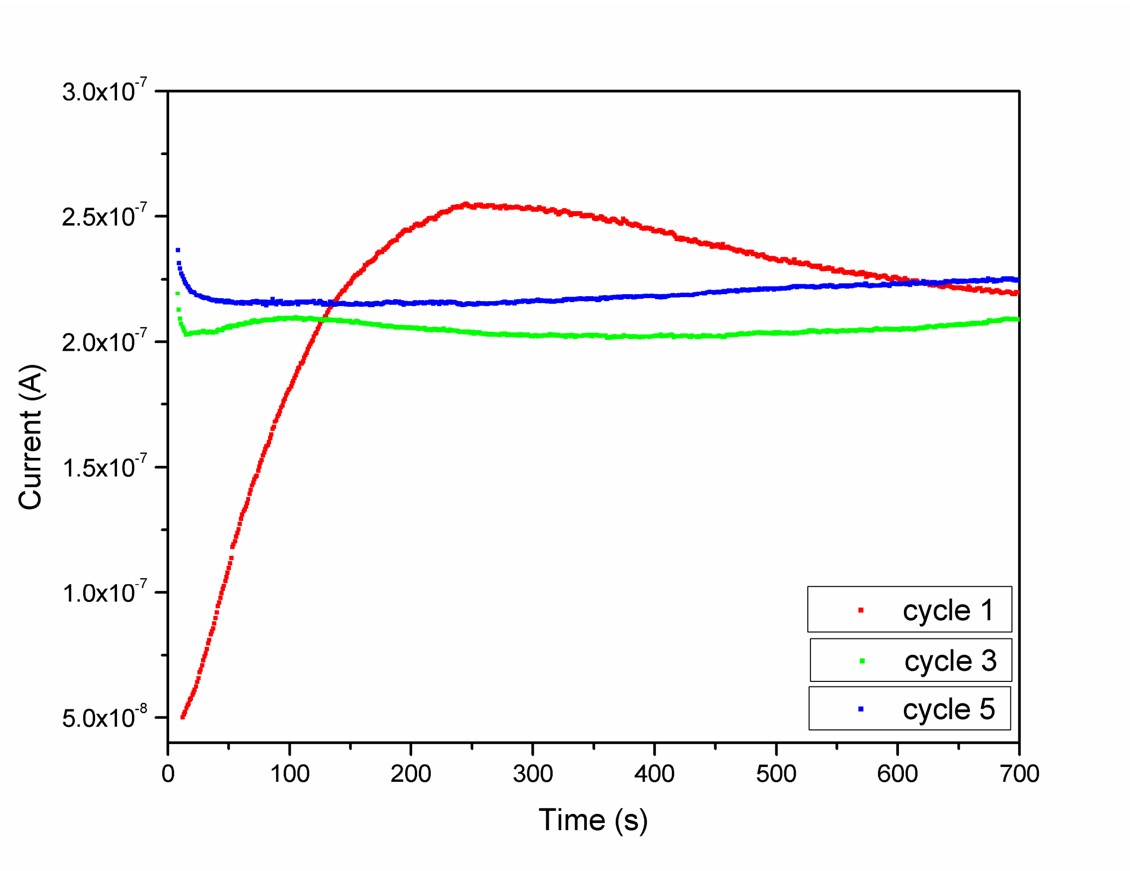

Figure S3. Current evolution during transport experiments for 1st, 3rd and 5th cycle.
